# Insufficiency Fracture in a Patient With Rheumatoid Arthritis in Remission Receiving Long-Term Methotrexate Without Conventional Risk Factors: A Case Report and Literature Review

**DOI:** 10.7759/cureus.107806

**Published:** 2026-04-27

**Authors:** Hanane Hajar, Achemlal Lahcen

**Affiliations:** 1 Rheumatology, Faculty of Medicine, Mohammed VI University of Health Sciences (UM6SS), Casablanca, MAR; 2 Rheumatology, Mohammed VI International University Hospital, Rabat, MAR

**Keywords:** fatigue fracture, methotrexate, mri, multidisciplinary management, osteopathy, rheumatoid arthritis

## Abstract

Methotrexate (MTX) is the standard care for rheumatoid arthritis (RA), and it is largely tolerated with a small percentage of patients (in long-term studies) discontinuing due to side effects. MTX-associated osteopathy is an infrequent complication, which is marked by insufficiency fractures during long-term therapy even in individuals with properly controlled RA. We report the history of a 75-year-old seropositive remittent-RA patient with a history of more than 18 years of MTX therapy, complaining of nontraumatic mechanical ankle pain that has progressively caused the patient to walk with a limp. Laboratory tests, including inflammatory markers, were normal, and there was no sign of disease flare. Magnetic resonance imaging demonstrated calcaneus and distal tibia stress fractures with significant bone marrow edema, and no active synovitis. MTX-induced osteopathy was also considered in a multidisciplinary setting, which resulted in MTX withdrawal and nonpharmacological treatment using off-loading, analgesia, and the optimization of bone-targeted therapy. The clinical history was positive, and the pain gradually improved during follow-up.

## Introduction

Methotrexate (MTX) remains the cornerstone of first-line treatment for rheumatoid arthritis (RA) according to current American College of Rheumatology and European Alliance of Associations for Rheumatology guidelines, due to its proven efficacy and favorable long-term safety profile at low weekly doses [1]. RA is classified as a chronic systemic inflammatory disease defined by persistent synovitis, progressive structural damage, and functional impairment.

Although the most frequently identified side effects of MTX consist of gastrointestinal, hepatic, and hematologic toxicities, uncommon skeletal complications have also been reported. MTX-associated osteopathy is an infrequent and likely neglected entity characterized by insufficiency or stress fractures in the lower extremities, specifically the tibia, calcaneus, and metatarsal bones [2]. While guidelines mandate strict monitoring for hepatotoxicity and cytopenias, skeletal health is often only monitored in the context of corticosteroid-induced osteoporosis, leaving MTX-associated bone toxicity as a significant diagnostic blind spot.

MTX-associated osteopathy initially became known in children receiving treatment for acute leukemia. This disease has been described as a combination of increasing bone pain, reduced bone density, and stress fractures [3]. The diagnosis can be especially challenging in people with RA, as bone pain and walking impairment may be incorrectly attributed to a disease flare. Furthermore, establishing a causal relationship requires excluding other metabolic bone diseases and mechanical factors, often necessitating a detailed assessment of causality.

We herein report the case of a 75-year-old woman with long-standing seropositive RA in sustained remission who presented with atraumatic calcaneal stress fractures while receiving long-term MTX therapy.

## Case presentation

A 75-year-old woman presented with a 12-month history of progressively worsening, nontraumatic mechanical pain involving the right ankle. Her medical history was notable for long-standing seropositive RA, diagnosed in 1995, which had remained in sustained clinical and biological remission for several years under MTX therapy at a dose of 15 mg weekly for more than 15 years, in combination with folic acid supplementation. While this is within the standard therapeutic range for RA, it is worth noting that, in an elderly patient, the cumulative exposure over 15 years may have contributed to skeletal toxicity, particularly if renal function fluctuates.

She also had a history of anxiety disorder treated with anxiolytic therapy and migraine. The patient reported intermittent use of vitamin D and calcium supplementation to prevent osteoporosis. The patient reported increased functional impairment caused by pain, including difficulty ambulating and the requirement for a cane when walking long distances. The symptoms were purely mechanical, worsening with weight bearing and improving with rest. Her body weight was 82 kg, and her height was 163 cm. She denied any severe trauma that preceded her complaints.

Clinical examination showed a localized ache over the right calcaneal region but no considerable swelling. The pain was mechanical in nature and aggravated by weight-bearing. While walking, the gait was analgic.

There was no hindfoot or midfoot deformity, nor was there any evidence of midfoot osteoarthritis or posterior tibial tendon dysfunction. No synovitis was observed in peripheral joints, including hands, wrists, knees, and the contralateral foot. The remainder of the physical examination was unremarkable.

**Figure 1:**
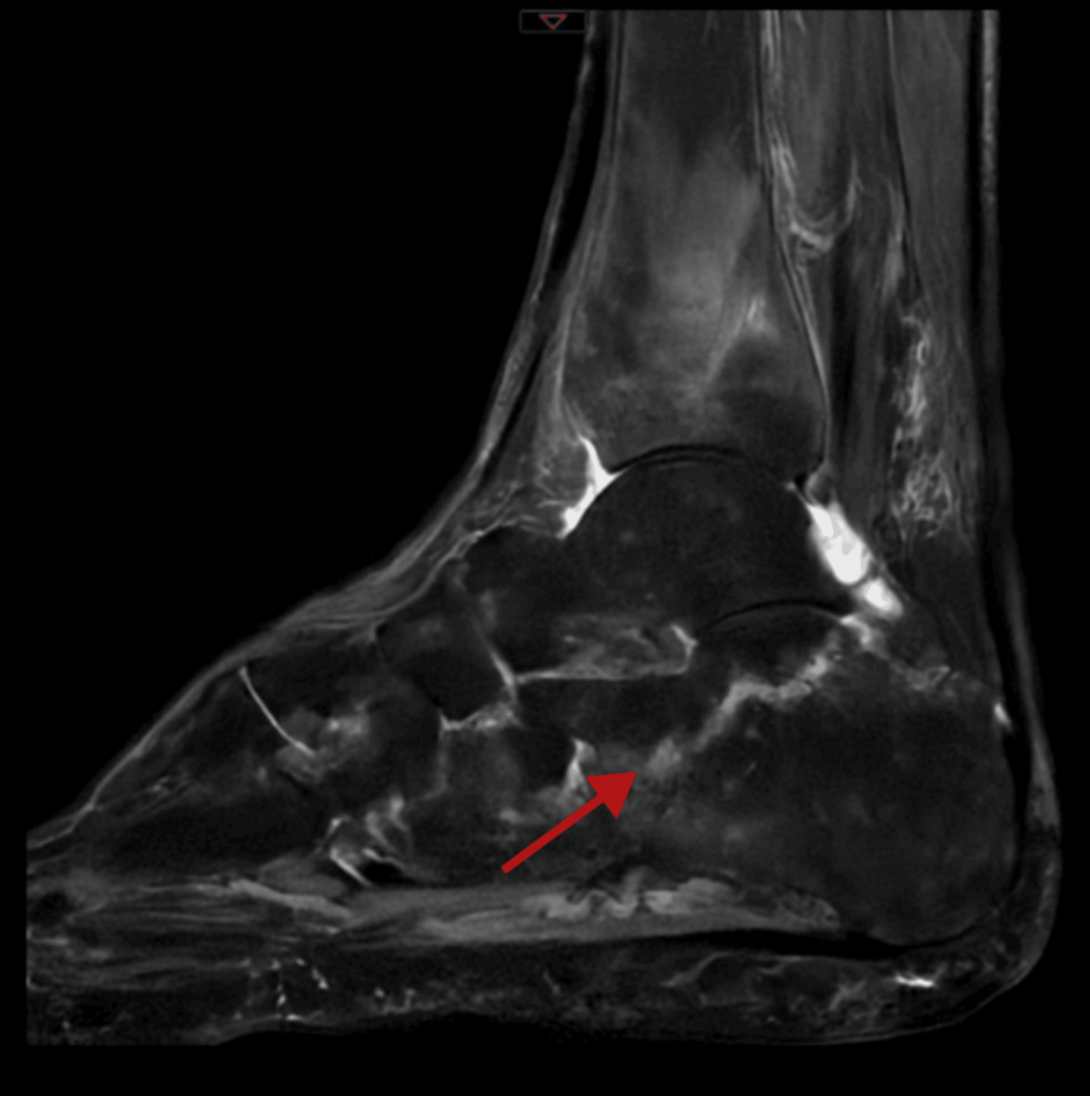
T2-weighted MRI of the right calcaneus showing bone marrow edema with features suggestive of a stress fracture (red arrow) MRI: magnetic resonance imaging

**Figure 2:**
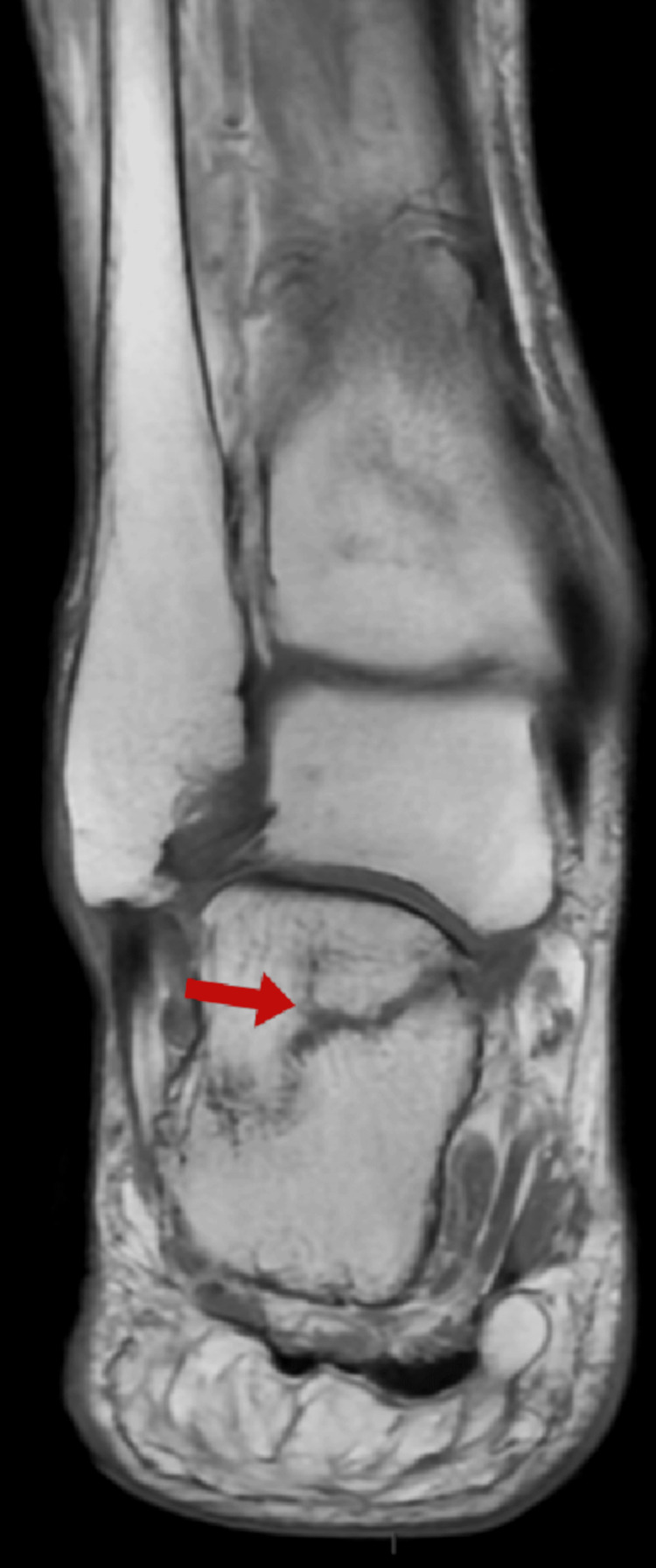
Sagittal T2-weighted MRI demonstrating fracture line of the calcaneus MRI: magnetic resonance imaging

Magnetic resonance imaging (MRI) of the hindfoot (sagittal view) revealed extensive intraosseous hyperintensity of the calcaneus on fluid-sensitive sequences, primarily affecting the posterior-inferior and mid-portion of the bone, indicating bone marrow edema, indicating a potential stress or insufficiency fracture line (Figures 1, 2). Radiographs of the foot were either unremarkable or revealed only minor or early alterations, demonstrating the superiority of MRI in detecting early insufficiency fractures.

Biological tests, including inflammatory markers, were within normal ranges (Table 1). Bone mineral density (BMD) measured by dual-energy X-ray absorptiometry (DEXA) showed no evidence of osteopenia or osteoporosis.

**Table 1:** Laboratory findings CBC: complete blood count; eGFR: estimated glomerular filtration rate; AST: aspartate aminotransferase; ALP: alkaline phosphatase

Category	Parameter	Result	Reference range	Unit
Inflammatory markers	C-reactive protein	Normal	<5	mg/L
	Erythrocyte sedimentation rate	15	<20	mm/hour
Serology	Rheumatoid factor	Positive	Negative	IU/mL
	Anti-cyclic citrullinated peptide antibodies	Positive	Negative	U/mL
Hematology (CBC)	Hemoglobin	12.8	12.0-15.5	g/dL
	White blood cell count	6.2	4.0-11.0	×10⁹/L
	Platelets	210	150-450	×10⁹/L
Renal and hepatic	Serum creatinine	0.8	0.6-1.1	mg/dL
	eGFR	>60	>60	mL/minute/1.73 m²
	ALT/AST	22/19	<40	U/L
Metabolic bone profile	Vitamin D (25-OH)	34	>30	ng/mL
	Serum calcium	9.4	8.5-10.2	mg/dL
	Serum phosphate	3.2	2.5-4.5	mg/dL
	Alkaline phosphatase	72	44-147	U/L
	Parathyroid hormone	38	15-65	pg/mL

Based on clinical and imaging findings and after multidisciplinary discussion, the diagnosis of MTX-associated osteopathy with insufficiency fractures was established. MTX was discontinued, and the patient was managed conservatively with limb offloading, analgesic treatment, and optimization of bone-supportive therapy.

Given the need to maintain disease control of RA, leflunomide (Arava®) was introduced as an alternative disease-modifying antirheumatic drug. In addition, intravenous bisphosphonate therapy with zoledronic acid (Aclasta®) was administered as part of bone-protective management. Clinical follow-up showed progressive improvement in pain intensity, with a reduction in visual analog scale score and gradual recovery of walking capacity.

## Discussion

MTX-associated osteopathy is an uncommon but probable consequence of MTX treatment in rheumatoid or psoriatic arthritis. Osteopathy was first reported with high-dose MTX used in acute leukemia, but it can also occur with long-term low-dose MTX used in inflammatory disorders. The clinical triad of pain, osteoporosis, and stress fractures of unusual placement is typically used to characterize this osteopathy [3].

The precise pathophysiological mechanism is still not fully clarified. Experimental data suggest that MTX interferes with osteoblast proliferation and differentiation, leading to damaged bone formation and delayed microdamage repair [4]. As shown by our case, this altered bone remodeling may predispose patients without overt osteoporosis to insufficiency fractures.

A point of consideration raised during clinical review is the MTX dosage. In this case, the patient was maintained on 15 mg/week, which, while standard for RA, must be carefully evaluated in an elderly patient. However, the patient’s renal function was well-preserved (estimated glomerular filtration rate >60 mL/minute/1.73 m²), and hematologic markers were normal (Table 1). Since MTX is primarily excreted renally, the absence of renal impairment suggests that the fractures were not the result of acute systemic drug accumulation, but rather the consequence of chronic, localized inhibitory effects on bone remodeling. As demonstrated by our case, this altered remodeling can predispose patients to insufficiency fractures even without overt osteoporosis.

Differential diagnosis in such cases includes insufficiency fractures secondary to postmenopausal osteoporosis, corticosteroid-induced bone fragility, and metabolic bone disorders. Our case stands out because the patient was in clinical and biological remission, had no history of corticosteroid use, and possessed a normal BMD on DEXA scan. These factors effectively narrow the differential and point toward MTX-associated osteopathy as the most plausible diagnosis.

To objectively formalize this relationship, we applied the Naranjo Adverse Drug Reaction (ADR) Probability Scale. The assessment yielded a score of 7, classifying the event as a "probable" ADR. This score was supported by the temporal relationship between long-term MTX use and fracture onset, the exclusion of alternative metabolic causes (such as vitamin D deficiency or hyperparathyroidism), and the significant clinical and radiographic improvement upon drug withdrawal (dechallenge).

A review of previously published cases shows that almost all of the described cases were elderly women with RA receiving long-term MTX therapy, often with confounding factors such as chronic corticosteroid use, low BMD, or persistent inflammatory activity [2,5]. Our case differs from these reports in a number of important ways. The patient was keeping clinical and biological remission, with no evidence of active synovitis and normal BMD on DEXA, limiting the differential diagnosis and increasing the possibility of MTX-associated osteopathy.

According to a review of previously reported cases, the majority of patients are elderly women with RA who are taking long-term MTX therapy, often with additional risk factors such as corticosteroid exposure, low BMD, or persistent inflammatory activity [6,7]. In contrast, our case stands out for the lack of disease activity and a normal DEXA scan, which helps to narrow the differential diagnosis and increases the likelihood of MTX-related bone toxicity.

The lack of trauma is a significant diagnostic clue. Chronic atraumatic mechanical pain in the hindfoot, especially in a patient on long-term MTX, should prompt immediate investigation for insufficiency fractures. MRI is the gold standard in this context; while initial radiographs are often normal, MRI is highly sensitive in identifying bone marrow edema and fine linear fracture lines, facilitating earlier diagnosis [8].

The significance of insufficiency fractures in rheumatologic patients has been highlighted by large case series and literature reviews, emphasizing the need for high clinical suspicion, particularly in patients with inflammatory arthropathies and those receiving bone-modifying treatments [9,10].

The Naranjo ADR Probability Scale was used in a formal causality assessment to determine whether MTX exposure and the incidence of insufficiency fractures are related. Based on the temporal relationship between long-term MTX use and the onset of symptoms, the lack of alternative causes like osteoporosis, trauma, or active inflammatory disease, and the clinical improvement seen following drug discontinuation, the assessment indicated a probable association. There was no ethical justification for the rechallenge.

From a therapeutic standpoint, most published cases highlight the benefits of discontinuing MTX in conjunction with offloading and symptomatic treatment [5]. In our patient, discontinuing MTX resulted in progressive improvement in pain and walking capacity, supporting the causal relationship. Overall, this case contributes to the growing body of evidence indicating that MTX-associated osteopathy can occur even in the absence of osteoporosis, emphasizing the need for increased clinical awareness of this uncommon complication.

## Conclusions

MTX-associated osteopathy remains a frequently overlooked diagnostic entity in the management of RA. While long-term corticosteroid therapy is a well-known contributor to fragility fractures, this case demonstrates that MTX can induce significant osteopathy and insufficiency fractures even in the absence of steroids and despite a normal BMD.

The diagnosis was supported by a "probable" causality assessment using the Naranjo probability scale, evidenced by the absence of alternative triggers and the rapid clinical and radiographic resolution following MTX discontinuation (dechallenge). Clinicians must maintain a high index of suspicion for this condition when a patient on long-term MTX therapy presents with chronic, localized mechanical pain. Because plain radiography often lacks sensitivity, magnetic resonance imaging (MRI) is the preferred tool for early identification.

Increased awareness of this "diagnostic trap" is essential to ensure that clinicians do not misinterpret these fractures as inflammatory flares or age-related osteoporosis. Timely drug cessation and skeletal offloading are critical to preventing diagnostic delay, avoiding unnecessary interventions, and improving long-term patient outcomes.
